# Fostering intergenerational education: An experiential learning program for medical students and older adults

**DOI:** 10.36834/cmej.69327

**Published:** 2020-09-23

**Authors:** Rebecca H. Correia, Lindsay Klea, Graham Campbell, Andrew P. Costa

**Affiliations:** 1 Department of Health Research Methods, Evidence, and Impact, McMaster University, Ontario, Canada; 2 Michael G. DeGroote School of Medicine, McMaster University, Ontario, Canada

## Implication Statement

Educational initiatives providing intergenerational, experiential learning opportunities can engage students of various education levels and disciplines. All persons can benefit from initiatives with older adults because our aging population suggests more of these interactions will occur across sectors. While students pursuing an education in health or medical fields are primarily identified as benefiting from intergenerational education to gain skills and knowledge to effectively care for the elderly, these teachings are invaluable regardless of one’s age, education, or career background. The program delivery and evaluation criteria can be adapted to assess competencies essential to different education or career paths.

## Déclaration des répercussions

Les initiatives en matière d’éducation procurant des occasions d’apprentissage expérientiel intergénérationnel peuvent favoriser la participation d’étudiants de différents niveaux de formation et de diverses disciplines. Toutes les personnes peuvent tirer avantage d’initiatives avec des adultes plus âgés, car notre population vieillissante suggère que plusieurs de ces interactions se produiront dans tous les secteurs. Alors que les étudiants poursuivant leur éducation dans des domaines de la santé et de la médecine sont principalement reconnus comme tirant profit d’une éducation intergénérationnelle pour acquérir des habiletés et des connaissances pour prendre efficacement soin des aînés, ces enseignements sont inestimables, peu importe l’âge, l’éducation ou l’expérience de carrière d’un individu. La prestation du programme et les critères d’évaluation peuvent être adaptés pour évaluer les compétences essentielles à divers parcours éducatifs ou professionnels.

As the Canadian population ages, more physicians with skills to care for the elderly are needed.^1^ The Canadian Resident Matching Service reports that only 3.3% of matched medicine subspecialty applicants identified geriatric medicine as their first choice discipline during the first match iteration in 2019.^2^ A lack of medical student exposure to older adults may be impeding an interest in caring for this demographic. Research demonstrates that experiential learning in medical curricula improves the relevance, attitudes, and retention of classroom material.^3^ Previous education programs involving clinically-oriented experiences with older adults have increased positive attitudes.^3^^,^^4^

The “Make a New Old Friend” program provides an intergenerational, experiential learning opportunity for medical students to increase competencies in caring for older adults, enhance communication skills, and explore career pathways in geriatric medicine. This program fosters mutual benefits for students and older adults by creating opportunities for intergenerational knowledge exchange and companionship.

The Waterloo Regional Campus of the McMaster University DeGroote School of Medicine recently developed and launched “Make a New Old Friend.” To date, eight medical students, eight older adults, and four geriatric specialists participated in two program iterations. Pre-clerkship medical students primarily enroll due to their geographic stability. The office of the Hamilton Integrated Research Ethics Board granted this program evaluation a waiver of ethical review.

We collaborate with staff at a Long-Term Care (LTC) residence to enroll older adults and pair them with students. Students and older adults meet in-person on a bi-weekly or monthly basis to facilitate relationship-building. Students and faculty mentors practicing geriatric medicine are matched to discuss their experiences and arrange shadow days.

Students complete an interest survey to facilitate the matching process. Onboarding packages are distributed and contain meeting guides with suggested conversation topics, communication tips, and meeting logs. Researchers facilitate a workshop for students about accommodating the personal expressions of older adults with cognitive impairments.

**Figure 1:**
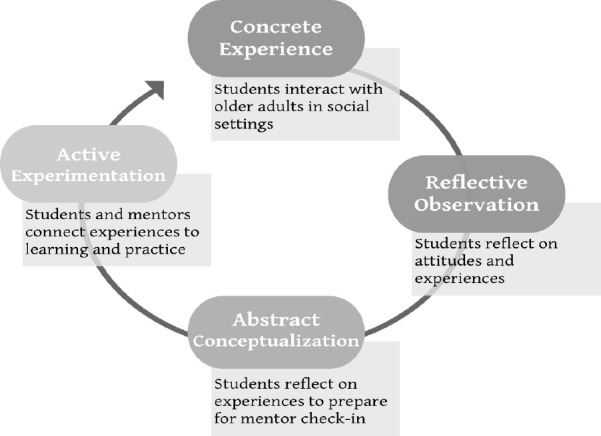
Examples of “Make A New Old Friend” experiences and reflections, adapted from Kolb’s Experiential Learning Model^5^

Students complete evaluations based on Kolb’s Experiential Learning Cycle to consolidate learning outcomes (see Figure 1).^5^ Mentors guide critical reflection based on students’ concrete experiences engaging with older adults, reflective observations, abstract conceptualization of experiences and knowledge, and active experimentation to consider influences on their education and future medical practice.

Program coordinators collect semi-structured feedback from participants. Overall, students report high levels of satisfaction with this program. Notable strengths include the regular meeting frequency to build rapport and develop communication skills. Reported barriers include administrative intake procedures and the structure of faculty mentor meetings. Suggestions for future iterations include streamlining intake and training procedures, providing more conversations topics and activities for meetings, facilitating subsequent geriatrics-focused clinical skills sessions, and engaging the older adult’s family in the program.

Program debriefs with management staff at the LTC residence evaluate collaborative program procedures. Staff identified high satisfaction among older adults regarding the nature of visits and students’ complete engagement during visits. The limited number of student participants, particularly male students, is a weakness to be addressed in future program iterations. Enhancing the evaluation rigour in the future may be possible with psychometric scales applied before and after exposure to experiential learning.
